# Gaps in sexual health content of healthcare professional curriculum: a systematic review of educational interventions

**DOI:** 10.1186/s12909-023-04901-1

**Published:** 2023-12-07

**Authors:** Nikole Bekman Troxman Prize, Sarit Shimony - Kanat, Anna C. Kienski Woloski Wruble

**Affiliations:** School of Nursing in the Faculty of Medicine, Henrietta Szold Hadassah Hebrew University, Jerusalem, Israel

**Keywords:** Sexual health content, Sexual health educational intervention, Sexual health assessment, Health professional students

## Abstract

**Background:**

Sexual health care, including assessment and patient teaching, is part of comprehensive patient care. Health professional (medical and nursing) students’ education in sexual health lacks uniformity in content and assessment skills.

**Objectives:**

The current systematic review aimed to assess sexual health educational curriculum for health professional students regarding the educational content, duration, and evaluation of these educational interventions.

**Methods:**

This systematic review followed the preferred reporting guidelines for systematic reviews. A comprehensive search was conducted between May–August, 2023 across four databases (PubMed, Scopus, CINAHL, EMBASE), outlining 614 sources. Following the screening process, 36 educational intervention studies were deemed eligible for inclusion. The quality assessment of these studies was conducted using The Effective Public Health Project tool, which was found appropriate for evaluating this type of research.

**Results:**

The studies had a global representation, with most studies conducted in the US. Limited nursing educational interventions were found. Three main categories emerged from the analysis of the educational interventions: one-time interventions, workshops, and semester courses. These categories differed in terms of their duration. Upon evaluating the educational intervention programs, it was found that the majority relied on participant self-reporting, while only a few included objective evaluations.

**Conclusions:**

This review revealed inconsistencies in educational content for healthcare professional students and may impact their clinical skills, particularly in sexual health. The variation in content, duration, and evaluation methods created challenges in assessing the interventions. The lack of standardized sexual health education highlighted a significant gap, raising concerns about students’ ultimate proficiency in this area. Bridging this divide is essential by integrating comprehensive sexual health content and assessment skills into the health professional curriculum.

## Introduction

The World Association for Sexual Health (WAS) emphasized sexual health (SH) as a fundamental right of every patient [1]. Incorporating SH into a patient’s overall well-being has been identified as an essential approach to delivering comprehensive health care and promoting overall health [2]. Moreover, it was considered an integral professional responsibility within health professional’s practice [3] and basic/advanced education [4]. The sexual health assessment (SHA), positioned as a primary step of the health evaluation process, serves as an informed framework for addressing SH issues [5]. Recognizing the integrated relationships between SH and all dimensions of life, a SHA allowed identifying existing or potential problems related to sexual well-being and potentially influencing the patient’s and family’s quality of life [5]. The results of a SHA enabled health professional students to develop a personalized treatment approach that aligns with the patient’s needs and preferences. SHA demanded a constructive perspective, encompassing positive attitudes, knowledge, and a proactive approach to initiate a conversation about SH [1, 6, 7].

The literature suggested that many healthcare providers lack the education and professional skills to address SH issues effectively. Studies such as the 2009 survey of American medical students [7] and the 2020 Danish national survey of health professional students [8] highlighted the persistent need for professional sexual health education (SHE) focused on acquiring SHA skills in the curricula. Students recognized the importance of SHA for patient health and well-being but also expressed a lack of appropriate training in this area. In studies that overall included over 1000 health professionals from diverse disciplines (medical and nursing), students report concerns about their unpreparedness regarding SH skills. Furthermore, the absence of SHA education in healthcare curricula can impact the ability of students to address SH issues in their future professional practice [9–13].

An SHA described as a part of a comprehensive health history, requiring integration of questions about SH. In order to conduct a SHA, it was essential to have relevant knowledge and communication skills [5, 14]. The National Coalition for Sexual Health (2021) recommended considering the following six aspects, the 6 P’s, during the patient’s SHA: partners, practices, past history of STI(s), protection, pregnancy and fertility, and P plus, which include pleasure, problems/dysfunctions, and Pride-LGBTQ issues [15]. One of the popular models for discussing sexuality with patients is the PLISSIT model [16]. It provides a delineated approach, beginning with introducing the topic by obtaining the patient’s permission to discuss personal SH.

Professional experts for undergraduate medical education agreed that integrating the principles of SHA (knowledge, attitudes, and skills) should be a mandatory component of SHE for professional practice [17]. Principles of SHA and 6 Ps’ should be integrated into the curricula as part of the essential first–year skills for assessing the SH of the patient. The second-year curricula should focus on more advanced SHA skills, such as understanding the impact of various diseases on SH function [17]. Though specific recommendations for studying SHA are not offered, the importance of having an objective structured clinical examination (OSCE) at the end of the first year and a theoretical summative exam at the end of the second year as a measure of the effectiveness of the curriculum was highly recommended [18]. While these recommendations were intended for medical students, it is essential to recognize their significance for all health profession students directly involved in patient care. It is important to note that in the context of nursing professional education, many SH topics are not included as compulsory content in the curriculum [10, 11]. This lacuna persisted despite detailed information on SHA steps in nursing textbooks [5].

The impact of SHE curricula on improving the healthcare providers’ ability to address patients’ SH has been described in the literature [19, 20]. A review of educational intervention studies published between 2002 and 2020 contains 11 studies that found evidence supporting the effectiveness of SHE in improving knowledge, attitudes, and skills despite variations in intervention duration and educational content [20]. However, only one study included senior nursing students, while the intervention curricula in the other studies targeted healthcare providers who had already graduated. Furthermore, the findings indicated significant variation in the duration and content of the interventions, with the majority not focusing on skill acquisition [20].

In contrast to previous results, another systematic review included 11 educational intervention studies in which the intervention focused on training SHA skills [19]. Only two studies used actual evaluation measures to examine training effectiveness on improvement in SHA actual performance (OSCE and simulations). However, the performance observed following the intervention was tested under laboratory conditions and not with actual patients. In addition, only one study measured the actual performance by asking the patient whether they were asked about SH. The rest of the studies examined the effectiveness of the intervention by participants self-reporting. It is important to note that SHA skills in these studies mainly focused on assessing risk for sexually transmitted diseases (HIV, AIDS, STI) and did not include comprehensive SHA and communication skills [19]. In addition, the quality assessment level of the studies included in the systematic review was suboptimal due to differences in research objectives, evaluation metrics, and the usage of varying questionnaires. Finally, similar to previous results, the interventions in this systematic review included only medical students or interns, with no mention of nursing students [19].

The present systematic review aimed to identify SH educational interventions and evaluations of these interventions designed for health professional students. The review intended to analyze these programs by examining their content, focusing on SH education, and investigating the taught frameworks or courses. It seemed to determine whether SHA was included and, if so, what assessment principles (knowledge, attitudes, and skills) were incorporated as educational objectives. The review also aimed to assess the duration of these educational interventions and evaluate the methods used for assessing SHA principles, presenting self-reported data vis a vis actual performance in clinical settings.

## Method

### Searching strategy

The current systematic review followed the PRISMA guidelines for reporting systematic reviews [21]. The research team followed a predetermined protocol based on PICO strategy and inclusion–exclusion criteria. Inclusion criteria encompassed intervention studies employing a pre-post intervention research design, focusing on SH education tailored for nursing and medical students. The inclusion criteria were peer-reviewed educational intervention design published in English between 2005–2023, with SHA educational content. The search was conducted across four databases (CINAHL, EMBASE, PubMed, and Scopus) with the assistance of a librarian. The search was conducted between May and August 2023 and included 614 studies transferred to the continuous screening phase.

The following search terms were used: TI (“Medical Student*” OR “Nursing Student*” OR “Health Professional Student*” OR “Medical Studies” OR “Nursing Studies”) OR AB (“Medical Student*” OR “Nursing Student*” OR “Health Professional Student” *” or “Medical Education” or “Nursing Education”), TI (“Sexual Educator*” OR “Sexual Health”) or AB (“Sexual Education*” or “Sexual Health” or “Sexual health assessment” or “Sexual health history”), TI OR AB curriculum. MESH or TSEZARIUS were used in relevant databases.

### Study selection and data extraction

**Fig. 1:**
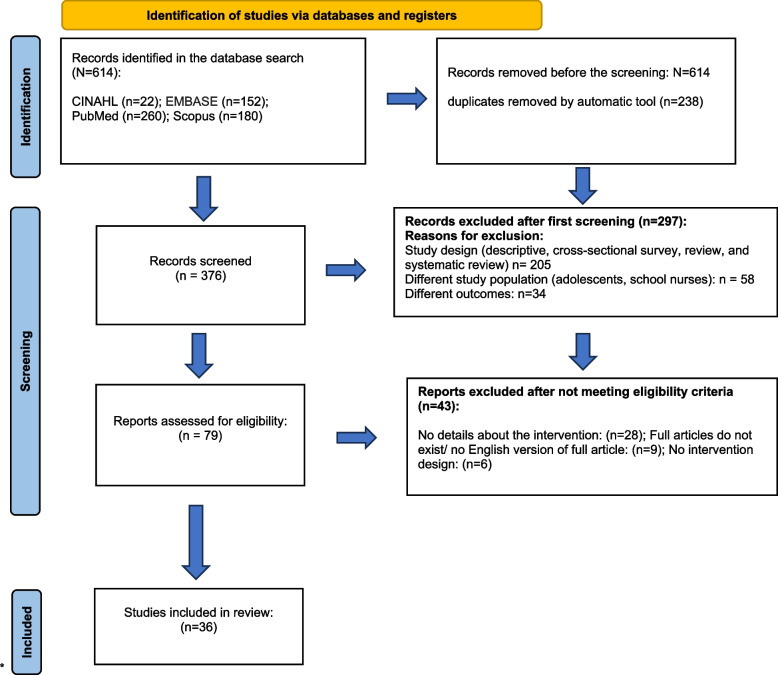
PRISMA 2020 flow diagram for new systematic reviews which included searches of databases and registers only. From: Page MJ, McKenzie JE, Bossuyt PM, Boutron I, Hoffmann TC, Mulrow CD, et al. The PRISMA 2020 statement: an updated guideline for reporting systematic reviews. BMJ 2021;372:n71. 10.1136/bmj.n71

During the study selection process, ZOTERO software was utilized for source management and duplicate identification. After removing duplicates, 297 studies were transferred to Rayyan software for sorting [22]. Initially, articles were screened based on their titles and abstracts, and only those aligning with the inclusion criteria (*n* = 79) were included in a second screening involving detailed reading. Eventually, after rigorous assessment, 36 articles meeting all criteria were included in the extraction stage (see Fig. 1). All phases of the screening process were independently conducted by two researchers (NB and AWW). The studies that presented conflicting decisions of the original two researchers were deliberated upon in a discussion involving the third researcher (SSK). Articles were included or rejected from the systematic literature review only after a consensus was reached through mutual agreement.

Data were extracted from the included studies based on the following criteria: study information (including the authors, year, and country of publication), study design, and target population. Information about sexual health education duration, content, assessment principles, and evaluation was offered.

### Quality assessment

The Effective Public Health Project (EPHPP) tool developed by Thomas et al. (2004) was utilized for quality assessment of studies included in the systematic review [23]. This tool has been extensively used in the literature and was considered valid for determining the quality of interventional research designs [24]. Each study was assessed based on the EPHPP tool’s seven components: selection bias, study design, confounders, blinding, data collection methods, intervention integrity, and analysis. In addition, the overall quality of each study was rated using a three-level scale (low, medium, and high quality) [23]. Two researchers (NB and AWW) initially performed an independent quality assessment. Subsequently, the findings were shared with the third researcher (SKK), who compared the results obtained by the two researchers. Finally, any discrepancies in the results were resolved through discussion and agreement.

## Results

### Study characteristics and target population

The present study involves a total of 36 research studies (*N* = 36) with a global representation- most of them (*n* = 19; 53%) were conducted in the US and involved medical students [25–43]. Of the studies reviewed, only 13 (36%) specifically targeted nursing. Among these, ten educational interventions involved nursing students [31, 37, 44–51], while the remaining studies were centered on professional nurses’ continuing education [36, 52].

Regarding study design, only 2 of the 36 (5%) studies utilized a randomized controlled trial (RCT) design [52, 53], with four additional studies (11%) employed quasi-experimental designs [44, 49, 51, 54]. Out of 30 remaining studies with pre- post test design, only seven (23.3%) conducted a follow-up (over different periods) after the intervention [25, 28, 35, 41, 44, 55, 56].

Most studies (*n* = 33; 92%) received a weak quality assessment (EPHPP = 3) due to selection bias, study design, and data collection procedures (see Table 1).

**Table 1:** Study characteristics

**Authors (Country)**	**Aim**	**Target Population (Sample, N)**	**Study design/Quality assessment**	**Intervention type: Lecture/group discussion/simulation/observation/online modules**
Cushing A, England, [55]	To evaluate the impact of an intensive short workshop on attitude, intention, and behavior relating SH communication	*N* = 219, fourth-year medical students	Pre-post intervention with one year of the cohort EPHPP^a^—2	The half-day workshop with interactive methods included a brief introductory lecture, small group discussion, role-play on four scenarios, and received feedback
Athanasiadis L, Greece, [57]	To test the effectiveness of an ED management workshop	*n* = 100(pilot study) *N* = 600 (*n* = 50/workshop, 12 workshops in total) Health professionals from five medical specialties	Pre-post evaluation EPHPP^a^—3	The 7-h workshop included four parts: Part one: online module Part two: small group workshop Part three: two presentations Part four: large group workshop
Gianotenn WL, Netherlands [56],	To evaluate the multidisciplinary rehabilitation team workshops (discipline-specific and team-specific training) for improving knowledge and communication skills	*N* = 302 rehabilitation professional staff	Pre-post evaluation with four months follow-up EPHPP^a^—3	Six sessions of three hours, with three weeks intervals. Various didactic methods were used, including lectures, discussions, role-playing sessions, simulation of cases, and simulation of team meetings. Homework and practice were included
Rosen R, USA, [25]	To evaluate the effectiveness of the intensive workshop on residents’ communication skills and management of sexual problems (Pilot study)	*N* = 34, residents from different disciplines	Pre-post evaluation, with six month’s follow-up EPHPP^a^—3	A half-day intensive workshop with interactive content
Loeb DF, USA, [26]	To evaluate the impact of the intervention on clinical practice	*N* = 25, medical residents	Pre-post intervention EPHPP^a^—3	Three-week intervention, consisted of didactic lecture and role-play scenario
Zabar S, USA [27],	To evaluate the impact of the intervention on communication skills, screening practice, and patient satisfaction	*N* = 21 health professionals from different disciplines	Pre-post intervention EPHPP^a^—3	Five 2-h workshops with 2–6-week intervals Interactive teaching methods included lectures, discussions, video demonstrations, and simulations
Wiskin C, UK, [53]	To evaluate the impact of the intervention on students’ attitudes toward STI patients	*N* = 299, second-year medical students	RCT EPHPP^a^—2	Rope-play activity and written vignette
Kennedy KM, Ireland, [58]	To evaluate the effectiveness of educational strategies addressing sexual violence	*N* = 105, third-year medical students	Pre-post test online evaluation EPHPP^a^—3	2-h interactive lecture
Sung SC, Taiwan, [44]	To evaluate the effectiveness of the intervention on students’ knowledge, attitudes, and self-efficacy related to sexual health care	*N* = 190, senior nursing students	Quasi-experimental with intervention and control group Pre-post evaluation with 5-week follow-up EPHPP^a^—2	12-week educational program with interactive methods, including lectures, teacher instruction, group discussion, role-play exercises, case analysis, value clarification, brainstorming, modeling, testing, visual media, pictures, reflection report
Kim JH, Korea [52],	To evaluate the effectiveness of the online intervention on sexual health competencies	*N* = 32 registered nurses	RCT with intervention and control group Post-test: 3 months after the intervention EPHPP^a^—3	The 8-week e-PBL methods consist of eight tutorials (2 h. each) with online discussion
Wang LY, USA, [28]	To evaluate the effectiveness of short sexual health training on the comfort level in female cancer	*N* = 110 health professionals from different disciplines	Pre-post test, with 3,6 months follow-up EPHPP^a^—3	Short training (30–45 min) included deductive methods of educating and communication skills training
Kim M, Korea, [45]	To evaluate the effectiveness of S-PBL on nursing students’ sexual knowledge. attitudes and gender role perception	*N* = 47 nursing students	Pre-post test EPHPP^a^—3	6 h. training program for all students S-PBL (4 h.) session only for the experimental group Different learning methods were used: didactic lectures, case study analysis, group discussion, a debriefing session
McBain L, New Zealand, [54]	To evaluate the effect of a simulation training program on performing clinical skills	*N* = 84 five-year medical students	A quasi-experimental, pre-post, and focus group evaluation (1-year cohort) EPHPP^a^—3	Online learning Practical session (4 h.)
Neff A, USA, [29]	To evaluate the effect of PBL^4^ on knowledge, attitude, and skills to care for LGBTQ, gender–nonconforming, and DSD community	*N* = 155 The first-year medical students	Pre-post test EPHPP^a^—3	One lecture and patient panel
Cooper MB, USA, [30]	To evaluate the effectiveness of LGBTQ training on students’ knowledge	*N* = 63 third-year medical students	Pre-post test EPHPP^a^—3	1 h. didactic lecture
Cummins AM, Australia, [46]	To evaluate the effectiveness of two workshops on improving personal and professional attitudes on sensitive topics	*N* = 53 first-year midwifery students	Pre-post survey test EPHPP^a^—3	Two interactive workshops First, at the beginning of the semester (before the clinical experience) Second, after the clinical settings
Micheal S, Australia, [59]	To evaluate the effectiveness of sexual health training on knowledge and general perspective	*N* = 121 third-year medical students	Pre-post test EPHPP^a^—3	2 h. session Flipped classroom method included: The online component (mini-lecture of 5–8 min before the session) Face-to-face session structure (5 topics)
Taylor LE, USA, [31]	To evaluate the Child Sexual Abuse Training Program on nursing students’ knowledge	*N* = 119 nursing students	Pre-post test EPHPP^a^—3	2 h. workshop with interactive teaching methods
Ross MW, Tanzania, [47]	To access the sexual confidence change following the intervention	*N* = 78 nursing and midwifery students	Pre-post test EPHPP^a^—3	2-day workshop on sexual health with the following interactive methods: Lectures, interactive sessions, dyads for sexual history-taking practice, small group discussion
Shroff S, USA, [32],	To evaluate the impact of the sexual history curriculum on students’ proficiency	*N* = 21 medical interns	Pre-post test EPHPP^a^—3	4 h. curriculum on sexual history taking on STI counseling Interactive teaching methods were used
Stumbar SE, USA [33],	To evaluate the impact of the intervention on increasing knowledge of LGBTQ sexual health	*N* = 90, second-year medical students	Pre-post test EPHPP^a^—3	2 h. intervention included a case-based lecture and patient panel
Frasca K, USA, [34]	To evaluate the impact of the intervention on HIV prevention knowledge	*N* = 34 internal medicine residents	Pre-post test EPHPP^a^—3	Two 4-h. online sessions
Salkind J, UK, [60]	To evaluate the effectiveness of compulsory training on improving knowledge on LGBTQ sexual health	*N* = 433, fifth-year medical students	Pre-post test EPHPP^a^—3	A half-day program included a lecture, patient panel, and clinal scenarios
Grova MM, USA, [35]	LGBTQ sexual health training	*N* = 27, general surgery residents	Pre – post-test, with 6 weeks of cohort EPHPP^a^—3	2-h. training included didactic methods and case scenarios
Natan S, USA, [36]	Trauma – informed training course	*N* = 21 APN nursing students	Pre – post test EPHPP^a^—3	2 – day course involved didactic methods and simulation
Ng AH, Hong- Kong, [48]	To evaluate the effectiveness of educational intervention in enhancing nursing students’ knowledge and attitudes regarding sexuality in older age	*N* = 222, first-year nursing students Intervention group = 169 Control group = 53	Pre-post test EPHPP^a^—3	7-h educational program consisted of one 3–5 lecture and 2-h group seminal presentations and discussions
Unal Toprak F, Turkey, [49]	To evaluate the effect of sexual health courses on the level of sexual reproductive health knowledge and myths	*N* = 130 third-year nursing students	Pre-posttest control group quasi-experimental study, with 1-week follow-up EPHPP^a^—3 (no sample size calculation)	Sexual health elective course, 14-week (42-h, curriculum) training program, 3 h. per week The course content was delivered in classroom format and consisted of the lecturer, student-prepared case studies, and video material
White BP, USA, [37]	To evaluate the effectiveness of comprehensive sexual health courses on improving knowledge, comfort, preparedness, and confidence level	*N* = 94, graduate nursing students	Pre – post test, with control group EPHPP^a^—3	Teaching methods included didactic quest lectures, clinical role-playing scenarios, videos, case studies, online discussions, small group discussions, and reflection
Friedlander R, USA, [38]	Evaluation of reproductive and sexual health curriculum on improving attitudes, comfort, and knowledge	*N* = 12 (classroom format) *N* = 23 (online format), medical physician assistant students	Pre- post test EPHPP^a^—3	The workshop consisted of five- sessions delivered twice: classroom and online setting
Mahabamunuge J, USA, [39]	To access the impact of a student-led lecture series on medical students’ comfort levels when dealing with diverse sexual health content	The study participants were graduated medical students from two consecutive academic years *N* = 847 (2018—2019) *N* = 862 (20,191—2020)	Pre – post-test EPHPP^a^—3	The seminar on “Gender and sexuality in medicine), consists of didactic lecture
Ojo A, USA, [40]	To evaluate a novel curriculum on students’ knowledge, comfort level, and skills about reproductive injustice	*N* = 68 senior medical students (third & fourth year)	Pre – post test EPHPP^a^—3	The 2-h session was delivered as a part of the broader mandatory Essentials II Medicine course of 4 weeks The intervention included a prerecorded video, an article, and interactive cases
Ross MW, USA, [41]	To evaluate the impact of long-term sexual health education on knowledge, counseling skills, and attitude change	*N* = 74, first-year medical students	Pre-post test with 16 weeks of cohort EPHPP^a^—3	The 1-semester course involved interactive teaching methods: didactic lectures, panels and tutorials, a video app with feedback, OSCE
Roth LT, USA, [42]	To evaluate the effectiveness of a long-term LGBTQ sexual health curriculum on changes in knowledge, comfort, and self-reported clinical impact	*N* = 70 medicine residents	Pre- post-test EPHPP^a^—3	One-year curriculum with interactive teaching methods: didactic and case-based sessions
Sarpkaya Guder D, Cyprus, [50]	To evaluate the effectiveness of sexual health course on students’ level of beliefs on sexual myths	*N* = 191 third-year nursing students	Pre-post semi-experimental study EPHPP^a^—3	Half of the course consists of face-to-face and online format
Bear MD, USA, [43]	To evaluate the effectiveness of a short online module lecture vs an in-class lecture on students’ attitudes and knowledge toward LGBTQ population	Year 1: *N* = 283 pharmacy students (online module) Year 2: *N* = 273 Pharmacy students (in-class module)	Pre- post-test EPHPP^a^—3	Online module and in-class materials
Mert – Karadas M, Turkey, [51]	To evaluate the impact of a practical communication educational program on students’ knowledge, attitudes, and ability to promote sexual health assessment	*N* = 48, 4^th^ year nursing students	Quasi-experimental (single group pre-posttest) study EPHPP^a^—3	The 8-week online educational program focused on the reproductive health of LGBTQ individuals and consisted of lectures, discussions, film display and analysis, sample video display and analyses, role-play activities, and standardized patient interviews

*Acronym:*^a^*EPHPP* Effective Public Health Practice, Quality assessment tool for quantitative studies: (degree of Q/A: 1-strong, 2- moderate, 3 – weak). Sources are presented in the table by year of publication; *SH* Sexual health, *ED*^*2*^ Erectile Dysfunction, *STI* Sexual Transmitted Infections, *e-PBL* Online Problem – Based Learning, *S-PBL* Simulation-based Problem Learning, *LGBTQ* Lesbian, Gay, Bisexual, Transgender, Queer, *DSD* Difference in Sex Development –affected, *HIV* Human Immunodeficiency Virus, *OSCE* Objective Structured Clinical Exam

### Intervention types

An analysis of the educational interventions revealed three main categories, namely one-time interventions (*n* = 9), workshops (more than 1-time intervention) (*n* = 20), and semester courses (*n* = 6) (see Table 2). Regarding one-time activities, most interventions (*n* = 7) concentrated on enhancing the SH of the LGBTQ population [29, 30, 33, 35, 40, 43, 59]. In two other studies, the educational interventions content focused on addressing particular issues, such as sexual violence [58] and sexually transmitted diseases (STD) prevention [53], with a SHA, adapted to the specific curricula content. There was no report on which principles and models were utilized to conduct a SHA in these training programs [53, 58]. The last study [54] centered on imparting clinical skills for conducting genital exams on both men and women. Again, the study solely focuses on practicing the technical aspect of the skill without incorporating an evaluation of SH.

**Table 2:** Characteristics of the educational intervention curricula, presented by types: one–time intervention, workshop, semester course

**Authors**	**Intervention Objectives**	**Instruments**	**Intervention components**	**Key findings**
**One-time intervention (only lecture/ practice session only)**				
Wiskin C, [53]	Attitudes Comfort level	25 items questionnaire included personal, perceived stigma, and social distance items	Role play with sexual health history-taking scenarios	No changes in a general attitude
Kennedy KM, [58]	Knowledge	No data about the questionnaire	One lecture	Knowledge improved after the intervention
McBain L, [54]	Skills Confidence Comfort levels	A questionnaire with closed and open questions No additional data about the instrument	Practical simulation session on male & female genital examinations	Skills, Confidence, and Comfort levels were improved after the intervention (*p* < .01^*^)
Cooper MB, [30]	Knowledge	The retrospective pre-post survey, 10-point rating scale	Didactic lecture	Knowledge increased in all domains, especially in the following: Knowledge about LGBTQ challenges health risks (pre: *M* = 5.8, CI: 5.4 -6.2) vs. (post: *M* = 8.1, CI: 7.8 – 8.4), (*p* < .01^*^) Knowledge about community resources for providing support to LGBTQ patients (pre: *M* = 3.7; CI: 3.1 – 4.3) vs (post: *M* = 8.1, CI: 7.5 – 8.7), (*p* < 0.4)
Stumbar SE, [33]	Comfort level	The online survey, eight items (1–5-point Likert scale)	Didactic lecture with three cases about: ✓ introduction to the social determinants of sexual and reproductive health of LGBTQ ✓ health disparities ✓ sexually transmitted diseases Interactive panel session with patients	All Comfort levels improved after the session. (*p* < .01^*^) with the most significant changes regarding: Discussing a patient sexual history (pre-mean rank = 14.5 vs post – mean rank = 18.9) Discussing sexual issues with patients > 60 y.old (pre- mean rank = 16.5 vs post mean rank (26.0) Treating patients with different sexual orientation (pre mean rank = 12.6 vs post mean rank = 14.6) Knowledge about the LGBTQ health concerns (pre mean rank = 15.1 vs post mean rank = 21.2)
Micheal MW, [59]	Knowledge	No data about the tool	The online material included the following topics: ✓ gender and sexual identity ✓ gender-based epidemiology ✓ gender as a social determinant of Health ✓ addressing health in a healthcare setting The interactive session consists of 5 stations (small group) with video clips and discussions on the following topics: ✓ Man’s/women’s/transgender health ✓ contact tracing for sexually transmitted infections ✓ gender and sexuality stereotypes	The average score for knowledge improved after the session in the following domains: gender and sexual health issues (6.3 to 8.2); women’s health services (5.7 to 7.6); men’s health services (4.4 – 6.9); sexual health services (5.8 – 7.6) (*p* < .01^*^)
Grova MM, [35]	Knowledge and skills Openness and Support Awareness of Oppression in the LGBTQ	AIM inventory, 19 items (1–5 Likert scale)	Training consists of five parts: ✓ Purpose of the training and educational objectives ✓ Allyship and Invitational Theory ✓ Strategies for Allyship and Intentionally Inclusive Care ✓ Case scenarios	Significant following effects were found: Knowledge: (*F* (1,7) = 8.30, *p* = .0024, ω^2^_p_ = .02 Openness: (*F* (1,7) = 6.14, *p* = .0042, ω^2^_p_ = .04 Awareness- *NS*
Ojo A, [40]	Knowledge Comfort levels toward reproductive justice (RJ)	No data about the questionnaire	4 cases on the following content: ✓ RJ & Indigenous health ✓ RJ & LGBTQ ✓ RJ & Maternal mortality ✓ RL & Family planning 25 min. recorded lecture	*n* = 28 (41%) – completed pre-test *n* = 31 (46%) -completed post-test *n* = 15 (22%) – completed both pre-and-post tests Following the intervention, participants reported enhanced levels of comfort and knowledge in certain subjects, though not across all areas No noticeable improvement in skills was observed after the intervention
Bear MD, [43]	Attitude Knowledge toward LGBTQ	ATLPS 5 point – Likert scale	Educational content was: ✓ Health disparities & terminology ✓ The basics of pharmacological endocrine treatment ✓ Gender identity assessment ✓	Year 1: *n* = 24 (8.5%) completed all survey Year 2: *n* = 141 (51.6%) completed both pre-and post-test Year 1: online module shows significant improvement in 1/9 survey items regarding the concerns of the LGBTQ population (*p* = .002^*^) Year 2: in-class materials: there was an improvement in 3/9 items after the intervention: The perceived competence to provide care (*p* = .02^*^) Competence to talk to LGBTQ patients (*p* < .001^**^) The belief that the curriculum addressed the PLBTQ population concerns (*p* < .001^**^)
**Workshops**				
**Authors (Country)**	**Intervention Objectives**	**Instruments**	**Intervention components**	**Key findings**
Athanasiadis L, [57]	Attitude toward patient-centered care Overall evaluation: new knowledge acquisition, quality of presentation, usefulness for clinical practice (5 = point Likert scale)	Patient–Practitioner Orientation Scale (PPOS), 18-item Cross Cultural Attitude Scale (CCAS), 29-item	Didactic components: ✓ Human sexuality, ✓ Principles of taking sexual history for ED patients, ✓ ED treatment and follow-up Workshops component: ✓ Role-play scenarios	Physicians’ Attitude (53.6% response rate) A significant difference in Attitudes after the intervention (PROS: *p* < .05^*^; CCAS: *p* < .01 ^*^) Overall evaluation (62.3% response rate) Tutorial sessions for “medical treatment of ED” (*p* < .001) ^**^ and role–play on sexual history taking (*p* < .05) * received higher evaluation scores
Rosen R, [25]	Communication skills Changing in clinical practice	The questionnaire constructed by the authors consisted of the following: 19 items (5-point Likert scale) for pre-intervention evaluation Six items (5-point Likert score) for post-intervention evaluation Six multi-choice questions for Follow-up	Faculty and patient-physician panel presentations about male and female sexual dysfunctions Patients and physician panels live interviewing	Pre-intervention evaluation: 88% of participants sometimes or rarely discussed sexual health issues with patients 48% reported low confidence in managing sexual problems in a clinical situation Post-intervention evaluation: 93% of participants reported that after the workshop, they could better identify common sexual problems 92% improved their communication skill in sexual history-taking Follow-up evaluation: (9/34; 28.2% response rate) 90% of participants ranked their comfort level in sexual health history taking greatly improved
Gianotenn WL, [56]	Communication skills	A Dutch version of KCAAS (knowledge, comfort, approach, and attitude toward sexuality)	The workshop content: ✓ Introduction ✓ Definition and theoretical framework ✓ Sexuality in the context of the Rehabilitation ✓ Staff’s attitudes and beliefs toward sexuality ✓ Sexual health history taking practice module ✓ Rehabilitation approach and treatment ✓ Review learning objectives for clinical practice. Conclusion and closing session (four months after the course)	Discipline-specific training: Documented improvements in knowledge and communication skills after the intervention (*p* < .001^**^), with no differences in follow-up evaluation Team-specific training: No results from pre-post evaluation Follow-up evaluation reveals improvement in knowledge (*F* = 16.00, *p* = .00), comfort (*F* = 8.8; *p* = .01^*^), and approach (*F* = 3.7; *p* = .05^*^)
Cushing A, [55]	Attitudes, behavioral intentions, and changes in actual behavior	Instruments constructed by Authors: Attitude scale (12 items, 5-item Likert scale), with face validity Behavioral Intentions – no data Actual behavior – no data	Didactic session based on the introduction of Sexual response Phases, and the PLISSIT model A practical session based on scenario analysis focused on identifying reasons for taking a sexual history, barriers, and strategies for improving the skills	Attitude change (87.6% response rate) Were significantly favorable changes in all 12 items after the intervention Behavioral Intentions (43.8% response rate) 34.7% of participants believed that they would ask about sexual health in their routine practice Actual behavior (one-year follow-up) with an 80.3% response rate; 92% asked patients about sexual health
Loeb DF, [26]	Practical skills in sexual health history taking	Chart review on the following domains: The rates of sexual history documentation The rates of specific components of the sexual history The effect of an intervention	Short (30 min) lecture about: ✓ The importance of sexual health history, ✓ The general principles of sexual health history taking Practical session (role-play scenario)	pre-intervention:369 charts reviewed post-intervention: 260 charts There were changes regarding the documented sexual health history component and the mean number of components A modest effect of the intervention was found
Zabar S, [27]	Skills and knowledge Clinical performance Patients satisfaction	OSCE (6 domains evaluation form) Chart review (5 items evaluation form) Survey (10 items). No additional data	The workshops focused on acquiring and practicing communication skills in the following areas: ✓ managing a different patient encounter ✓ screening and assessment for depression and alcohol use ✓ taking a sexual history; behavior change counseling	Skills and knowledge (*N* = 15) A significant change between pre-post intervention was found in the following domains in the overall communication (*p* = .004^*^) Knowledge = *NS* Clinical performance The screening rate improved depending on the clinical setting Patients’ satisfaction – *NS*
Kim JH, [52]	Primary outcomes measures: Evaluating SHC competencies (knowledge, attitudes, practice) Second outcome: nurses’ satisfaction	Sexual Health Care Knowledge Scale (33 items on a 2-point scale) Sexual Health Care Attitudes Scale (17 items on a 3-point Likert scale) Sexual Health Practice Scale (21 items on a 2-point scale)	The web-based interactive program consisted of short videos (3–5 min) on five cases: ✓ breast cancer ✓ endometrial cancer ✓ prostate cancer ✓ testicular cancer ✓ colorectal cancer	Knowledge level was significantly higher in the intervention group (*U* = 68.50, *p* = .041), 3 months after the intervention No difference in the attitude score (*U* = 68.50, *p* = .021) and the practice score (*U* = 155.50, *p* = .06) 52.9% of participants were satisfied with e-PBL intervention
Wang LY, [28]	comfort level and self-reported frequency of addressing cancer-relating sexual issues	Online survey Pre-test: 8 items on a 5-point Likert scale Post-test survey: seven items on a 5-point Likert scale	The theoretical part consists of: ✓ Introduction to the bio-psycho-social aspect of sexuality ✓ breast cancer-specific issues relating to sexuality and quality of life Practice part: a sexual health assessment technique “Did you CARD her?”	Significant improvement in the comfort level (*p* < .001^**^) and in the frequency of addressing sexual health (*p* < .001^**^) after the intervention
Kim M, [45]	Sex-role perception Sexual knowledge Sexual attitude	Gender–role perception scale 15 items (1–5 points Likert scale) Knowledge scale, 40 items in seven domains (2-point scale) Sexual attitude scale with 16 items (1–5 point Likert scale)	The first session included five lectures on the following topics: ✓ Human sexuality ✓ Social, Cultural, and Historical aspects of sexuality ✓ Human & Sexual subjects ✓ Health risk & unprotective sexual behavior ✓ Contraceptive methods The second session included S-PBL	Sexual knowledge significant improved in the experimental group (*M* = 0.83; *SD* = 0.08 preintervention vs. *M* = 0.92; *SD* = 0.06 postintervention) compare to control group (*M* = 0.83; *SD* = 0.09 pre-intervention vs. *M* = 0.84; *SD* = 0.08, postintervention), (*p* < .05^*^) Sexual attitudes changes were found in the intervention group (*M* = 3.66; *SD* = 0.26, pre vs. *M* = 3.79; *SD* = 0.26 post) compare to control group (*M* = 3.63; *SD* = 0.28, pre vs*. M* = 3.54; *SD* = 0.20 post), (*p* < .05^*^) Gender and Role Perception—*NS*
Neff A, [29]	Knowledge Attitudes Skills	Multiple choice questionnaire	Introduction lecture Patient panel and discussion	The posttest score was 20% higher compared to the pretest (range of 90%—99% correct answer vs. 29%—94% in the pretest)
Taylor LE, [31]	Knowledge	Multiple choice test (7 items)	Video materials and discussion	The mean score after training improved (91.9%) vs. the mean score before the training (45.5%) (*p* < .01^*^)
Cummins AM, [46]	Knowledge Confident	13-item survey (1–5 point Likert scale)	The first workshop focused on the sensitive issues in midwifery practice and included interactive games The second workshop is equipped with mindfulness techniques and the PERMA model	Confidence level and knowledge improved after the workshop (*p* < .05^*^)
Ross MW, [47]	Knowledge Communication skills Attitudes	Sexual health education professional scale (SHEP) (1–7-point Likert scale)	Sexual health workshop with sexual history taking practicing session	Improvement in all domains of knowledge and communication skills (p < .01^*^) Change in Attitude was not for all domains
Shroff S, [32]	Knowledge Attitudes	Case-based questions Likert scale questions No specific data about the tools	The curriculum included three introductory components: ✓ Flipped classroom ✓ Introductory communication skills ✓ Practice session of taking a sexual history	Mean knowledge score improved from 59% to 76T% (pre-post test) (*p* = .004) Median Comfort score for sexual history taking improved from 3.8 [QR v3.0, 4.0] to 3.8 [QR 3.6, 4.6] and 3.8 [QR 3.6, 4.0] to 4.1 [QR 3.9, 4.1] for male patients (*p* = .05) and female patients (*p* = .008) respectively Median frequency score for taking sexual history improved from 2.9 [QR 2.7, 3.0] to 3.1 [QR 2.8, 3.4] and 3.2 [QR 2.8, 3.7] to 3.4 [QR 3.2, 4.0] for male (*p* = .16) and female patients (*p* = .008) respectively
Frasca K, [34]	Comfort level	Self-assessment questions constructed by authors	Curriculum components: ✓ LGBTQ terminology ✓ Inclusive sexual history taking ✓ LCBTQ and HIV- related health disparities ✓ HIV risk assessment and prevention counseling ✓ PrEP candidacy and care delivery	Comfort levels increased after the training in all HIV prevention topics (*p* < .05^*^)
Salkind J, [60]	Confidence	self-assessment questionnaire constructed by authors	Training topics included: ✓ introduction to LGBTQ patients (lecture) ✓ interactive panel with LGBTQ patient ✓ seminar work with clinical–based cases	Confidence using appropriate terminology to describe sexual orientation increased from 62% (58 – 67%, pre) to 93% (91 – 95%, post), (*p* < .001^**^) and gender identity from 41% (36 – 46%, pre) to 91% (88 – 93%, post) (*p* < .001^**^) Confidence in the clinical assessment increased from 75% (71 – 79%, pre) to 93% (90 – 95%, post) (*p* < .001^**^)
Natan S, [36]	Knowledge Awareness Attitude	Self-assessment questionnaire constructed by authors (1–5-point Likert scale)	The didactic lecture included following topics: ✓ Trauma-informed care overview ✓ Sex trafficking ✓ Evidence-based practice for STI prophylaxis in sexual assault care ✓ Relationship-Centered Communication ✓ Strangulation ✓ Trauma-informed pelvic examination ✓ Consent and confidentiality Simulation in two clinical cases	Self – reported Knowledge, Awareness, and Attitudes were improved after the course (*p* = .01, 95% CI)
Ng AH, [48]	Knowledge and attitude	Aging sexuality knowledge and Attitudes Scale (ASKAS) consisted of a knowledge subscale (35 items) with a 3-point scale and an attitude subscale (26 items), with a 7-point Likert scale	Educational topics: ✓ Perspectives of love ✓ Sexuality of older adults ✓ Sexual dysfunction of older adults ✓ Chronic illness and sexuality of older adults ✓ Sexual coping of older adults ✓ Meaning of sexuality older adults ✓	the intervention significantly enhanced students’ knowledge (*F* (1,28) = 257.10, *p* < .001^**^), with Cohen ď of 3.7, and positive attitudes (*F* (1,128) = 51.17, *p* < .001^**^) with Cohen ď of 1.2
Friedlander R, [38]	Knowledge Comfort Attitude	Online, 4-point Likert scale survey designed by authors	SRH topics included: ✓ Diversity of sexuality and sex practices ✓ Sexual complaints across various populations ✓ Diversity in reproductive choice ✓ Options for unintended pregnancy ✓ Disparities in the peripartum period	Knowledge score improved from 54% (pre) to 60% (post) Comfort with taking a sexual history and discussion SRH increased: 0.92 for the classroom format and 0.50 for the online Attitude – NS
Mert – Karadas M, [51]		The attitude scale toward LGBTI consisted of 28 items, a 5-point Likert scale The Reproductive health history skills checklist has 38 questions (3-point scale) The effective communication skills evaluation form has 33 questions (3-point scale) Knowledge test, 16 multiple choices with 3 open-ended questions	Educational content consisted of: The theoretical part of Five modules: Basics concept, sexual orientation, the status of LGBT in the world and Turkey, reproductive health, and nursing care Practical part: simulations	the study found a significant increase in the median student’s knowledge (*p* < .001^**^), positive attitudes (*p* < .001^**^) and effective communication scores, and recording sexual health history (*p* < .001^**^) after the intervention
**Semester course**				
**Authors (Country)**	**Objectives**	**Instruments**	**Intervention components**	**Key findings**
Sung SC, [44]	Knowledge Attitude Self-efficacy	Knowledge of sexual healthcare Scale (31 items, 2-point scale) Attitude to sexual healthcare Scale (18 items, 5-point Likert scale) Self-efficacy for sexual healthcare Scale (22 items, 5-point Likert scale)	The educational program covered three main subjects: ✓ Bio-psycho-social aspect of sexuality ✓ the bio-psycho-social effect of illness, disability, and medical treatment on sexual problems ✓ communication skills and principles of sexual health history-taking	Significant differences were found in the intervention group relating to knowledge increased (β = -0.27; *P* < .001); attitude (β =—0.38, *p* < .001^**^), and self-efficacy (β =—0.90, *p* < .001^**^)
Unal Toprak F, [49]	Sexual health knowledge and Myths	The Sexual knowledge about reproductive sexual health (40 questions), 1/0 scale The sexual myths form – 46 expressions, True/False scale	Course content: ✓ Concepts related to sexual health & sexuality ✓ Physiological, Psychological, and Sociological factors affecting sexual health ✓ Reproductive health & sexual rights ✓ Physiology of sexual activity ✓ Sexual function disorders ✓ Sexual development across the lifespan ✓ Abnormal behaviors toward sexuality ✓ Sexual abuse & Sexual violence ✓ Sexual Health education & nursing approaches ✓ STIs & protection methods ✓ Sexual health problems in the LGBTQ community Sexual health problems in cancer patients	There was a statistically significant difference (*p* < .05^*^) between the intervention and control groups in terms of reproduction sub-dimensions of Sexual Knowledge Test There was no statistically significant difference (*p* < .05) ^*^ between the intervention and control group pre-post test mean scores regarding Knowledge and Myths
White LY, [28]	Knowledge Overall preparedness Comfort Confidence	36 items questionnaire based on TPB theory	Course content: ✓ Values reflection and provider self–inventory ✓ Health policy ✓ Clinical care of sexual minorities ✓ Clinical care of gender minorities ✓ Overview of global care ✓ HIV/AIDS, STIs including prevention, testing, and treatment ✓ HPV-associated diseases ✓ Comprehensive sexual history–taking, Risk Reduction ✓ Aging, mental health, human trafficking ✓ Emerging and re-emerging infectious diseases	Knowledge – NS Overall preparedness – improved in the intervention group after the intervention (*MD* = 1.50, 95% CI -1.03 – 1.97], *p* < .05^*^) Comfort – increased after the intervention regarding discussing sexual health with transgender persons (*MD* = 1.43, *p* < .05^*^), sexual trauma (*MD* = 1.10, *p* < .05^*^), older adults (*MD* = 1.17, *p* < .05^*^), and different sexual orientation persons (*MD* = 1.10, *p* < 0.5^*^) Confidence – confidence improved in the intervention group regarding describing sexual health disparities associated with sex workers (*MD* = 1.73, *p* < .05^*^), racial minorities (*MD* = 1.63, *p* < 0.5^*^), and in performing sexual health assessment for transgender patients (*MD* = 1.63, *p* < .05^*^)
Mahabamunug J [39],	Enhancing student comfort	based on a previous study, the anonymous questionnaire consisted of quantitative and qualitative questions No data was found about the reliability and validity	Seminar’s lectures content: Year 1: 2018 – 2019 ✓ Birth control & Family planning ✓ Geriatric & Palliative approach to the LGBTQ population ✓ Perspectives from intersex patients ✓ HIV prevention: PEP & PrEP ✓ Female genital cutting ✓ Care for the transgender adolescent ✓ Innovative practices for LGBTQ health center ✓ Elective termination of pregnancy & marriage management Year 2: 2019 – 2020 ✓ Birth control & Family planning ✓ Puberty suppression in transgender children ✓ Working with victims of domestic violence & human trafficking during COVID-19 ✓ Breast cancer and HIV stigma in immigrant populations ✓ STDs & stigma ✓ Trauma & PTSD	*n* = 152 (17.7%) – completed pre- test *n* = 105 (12.3%) – completed post-test The post-test result reveals improvements in student’s self -assessed comfort levels in following domains: Communicating with diverse patient population: Adult—(90% vs 59%) Adolescent- (83% vs 51%) Trans—(68% vs 29%) LGB—(84% vs 49%) Communicating about sexual health content: Termination of pregnancy—(73% vs 51%) Sexual violence – (65% vs 33%) Contraception options – (93% vs 78%) Medical transition – (57% vs 22%) Identifying female genital cutting—(44% vs 11%) Counseling patients on PrEP- (70% vs 27%)
Roth LT, [42]	Knowledge Comfort Impact on clinical practice	Knowledge assessed by 8 multiple choice question Comfort level and impact on clinical practice assessed by tools constructed by authors (1–5 points Likert scale)	Course topics included: ✓ Community LGBTQ resources ✓ Pre-exposure prophylaxis ✓ Introduction to LGBTQ health; HIV ✓ LGBTQ health for outpatient pediatricians ✓ Gender-affirming care ✓ Evidence supporting gender translation ✓ Communication and cultural components of sexual health	Knowledge increased from 25.2% correct answers to 38.5% (*p* = .01^*^) Comfort level increased: asking about sexual orientation (3.5 – 3.8, *p* = .02), gender identity (3.5 = 3.8, *p* = .02^*^), and sexual practice (3.4 – 3.8, *p* < .01^*^) Impact on clinical practice score increased for the intention domain (4.5 of 5), importance (4.8 of 5), and satisfaction (4.5 of 5) domains
Ross MW, [41]	Knowledge Attitudes Communication skills	SHEPS questionnaire with 26 items (1–7 Likert scale)	The theoretical part included the following topics: ✓ Sexual dysfunction and relationship ✓ Gender spectrum ✓ Sexual History Taking ✓ Female and Men sexual health and dysfunction ✓ Contraception ✓ Sex trafficking ✓ Abortion ✓ Child sexual abuse ✓ Chronic illness and Sexuality ✓ Sexuality and Disability The practical part consisted of 3 OSCE stations: ✓ female SP ✓ male SP ✓ transgender assigned male at birth SP	Knowledge and communication skills- a significant change was found with a high effect size. However, the differences between means scores in the pre-post-intervention were small, especially: Knowledge to discuss with young patients (from *M* = 3.59, *SD* = 2.19 to *M* = 1.93, *SD* = 1.17, *d*^*c*^ = 0.95); with middle age patients (from *M* = 4.12, *SD* = 2.30 to *M* = 2.15, *SD* = 1.29, *d*^*c*^ = 1.07); with older patients (from *M* = 4.96, *SD* = 2.16 to *M* = 2.63, *SD* = 1.43, *d*^*c*^ = 1.27), and with patients with sexual problems related to medical or surgical treatment (from *M* = 5.42, *SD* = 1.99 to *M* = 2.85*, SD* = 1.51, *d*^*c*^ = 1.46) Communication skills improved: with young patients (from *M* = 2.97, *SD* = 1.28 to *M* = 1.77, *SD* = 0.93, *d*^*c*^ = 1.07); middle age patients (*M* = 3.74, *SD* = 1.66 to *M* = 2.00, *SD* = 0.92, *d*^*c*^ = 1.30); with older patients (from *M* = 4.55, *SD* = 1.71 to *M* = 2.41, *SD* = 1.02, d^c^ = 1.48) Attitude revealed minimum change The correlation of the OSCE total with the attitude scale was *r*_*s*_ = -0.15, *p* = .21

*Acronym: LGBTQ* Lesbian, Gay, Bisexual, Transgender, Queer, *AIM Inventory* Ally Identity Measure, *ATPS* Attitudes towards Lesbian Gay Bisexual Transgender Patients Scale, *ED* Erectile Dysfunction, *OSCE* Objective Structured Clinical Examination, *SHC* Sexual Health Care, *e-PBL* Online Problem- based Learning, *S-PBL* Simulation – based Problem Learning, *PERMA* Positive emotion, Engagement, Relationship, Meaning and Accomplishment, *PrEP* Pre-exposure prophylaxis, *HIV* Human Immunosuppressive Virus, *STI* Sexual Transmitted Infection, *SPH* Sexual and Reproductive Health, *TPB* Theory of Planned Behavior, *SP* Standardized Patient, *M* Mean, *SD* Standard Deviation, *CI* Confidence Interval

^*^p < 0.05; ^**^p < 0.01

In the second category of educational interventions, 20 workshops were identified. Of these, only three studies utilized the PLISSIT model [16] or 6P’s component [15] for SHA. SHA in these studies also focused on specific educational topics [26, 55, 56]. For instance, SHA was taught according to the PLISSIT model [55, 56] yet specifically tailored for the rehabilitation department [56] or dermatological and rheumatological assessment [55]. In another study, SHA is based on the principles of the 6P’s, but there was no reference to the PLISSIT model [26].

The remaining workshop interventions (*n* = 17) incorporated SHA as part of the general educational topic, though the PLISSIT model is not mentioned. For example, two educational interventions focused on assessing sexual dysfunction in men and women [25, 57]. Three others concentrated on women’s sexual and reproductive health, including SHA for unplanned pregnancies, STDs, and pelvic infections [32, 38, 45], as well as SH in the elderly [48]. Another two studies focused on oncological topics, with one study basing their SHA on the “Did you CARD her?” model [28]. Another four educational interventions included SHA as part of their curricula, but SHA was not the primary focus, with no information on the PLISSIT model [27, 46, 51, 59]. Additional three studies focused on the SH of the LGBT population with no specific information regarding a model for SHA [34, 51, 60]. Finally, two educational interventions dealt with sexual trauma and child abuse but did not provide specific details on SHA [31, 36].

The final category of educational interventions included semester courses for “long-term training” (see Table 2). Of the four studies (*n* = 6), only one provided a comprehensive SHE curricula including advanced SHA skills, such as the effects of diseases/medications on sexual functioning [44]. The educational content in this study covered the biopsychosocial components of SH but did not specify which SHA model was used.

In the other five studies, SHA is adapted to the interventional topics and restricted to assessing LGBTQ SH [37, 39, 42], sexual reproductive health [49], and sexual dysfunctions [41].

### Duration of educational intervention

There was a wide variation concerning the duration of the educational intervention. For instance, the length of a one-time intervention curriculum ranged from a brief one-hour lecture [29, 30] to extensive training that entailed four hours of hands-on practice [54]. Most one-time educational intervention programs (*n* = 5) lasted two hours and utilized various teaching methods [33, 35, 43, 58, 59]. Three studies did not report on the duration of educational interventions [29, 40, 53].

Different durations of workshops were found across studies, ranging from 45 min [28] to 2 h [31], and 4 h [32]. Some interventions lasted half a day without specifying the exact hours [25, 55, 60], while others spanned between 6 to 8 h [34, 45, 57]. Longer interventions ranged from 10 h [27] to 16 h [52], 18 h [56], and two days, with no specific hour reporting [36, 47]. In two studies, no information was provided regarding the duration of the intervention program [27, 46].

Long-term educational interventions were different regarding duration, ranging from courses lasting a semester [37, 41, 44] to one annual course [41, 49] and a continual seminar without a precisely reported duration [39]. These educational interventions were delivered in the context of health professional educational curricula or professional staff educational interventions.

### Evaluation of the sexual health education curricula

Table 3 represents the analyses of SHE curricula (*n* = 11), including methods of evaluating the improvement in SHA performance following the intervention. Of these, eight studies revealed improvement in SH skills, but these findings were based solely on self-reports [25, 28, 34, 37, 42, 47, 54, 55]. Only three studies evaluated SHA using actual behavioral change [26, 27, 51]. One study by Loeb (2010) described improvement in SHA skills in professional practice based on patient chart review. After the educational intervention, participants frequently asked about sexual practices, partners, and contraception [26]. Another two studies [27, 51] used an OSCE clinical examination and chart review to evaluate the education curriculum. The study results indicated significant behavioral change regarding SHA and patient education in general. However, participants from one study [27], experienced only one simulation focused on SHA skills. In contrast, in another study, the SHA was evaluated online without follow-up [51].

**Table 3:** Evaluation of sexual health educational interventions

**Authors**	**Target Population**	**Intervention Objectives**	**Methods**	**Behavioral change outcomes**
Rosen R, [25]	Senior residents	Confidence in taking a SHH	Self-reported evaluation	comfort levels regarding obtaining a sexual health history—90% improved managing sexual health problems—56% improved
Cushing A, [55]	Fourth-year medical students	Attitudes, Intentions, Behavior changing	Self-reported evaluation, one-year follow-up	Out of 92% of participants reported behavioral change with proportional distribution among clinical settings: Obstetrics & Gynecology (82%) and Infectious disease (75%) were the most asked settings, while Internal medicine (18.4%) and Surgical (8.8%) were the most minor reported settings
Loeb DF, [26]	Medical residents	Rates of SHH and specific component documentation	Chart reviewed	Rates of sexual health history documented were improved after the intervention (*p* < .01^*^) The mean of sexual health-specific components increased after the intervention (*p* < .01^*^), with more frequent documented components of: Current sexual activity (17.1% pre vs. 20.7% post-intervention) Number of current partners (12.7% pre vs. 16.9% post-intervention) Sexual behaviors (0.8% pre vs. 1.5% post) Contraception (4.9% pre vs. 6.5% post) History of sexually transmitted infections (4.6% pre vs. 7.3% post) Issues with sexual performance (2.4% pre; vs. 6.5% post) History of abuse (1.36% pre vs. 2.3% post)
Zabar S, [27]	Health professionals	Communication skills Clinical performance	OSCE (pre-post test), only one simulation on SHH taking Chart review(pre-post)	Pre-post changes found in communication follow domains: (not specific for sexual health skills) Data gathering (*p* = .003) Rapport building (*p* = .001) Patient education (*p* = .02) Clinical performance (only one question was specific to sexual health) Improvement was found in the question about the current patient activity (*p* = .002)
Wang LY, [28]	Health professionals	Frequency of addressing cancer- relating sexual health issues	Self-reported survey	Significant improvement in the frequency of addressing sexual health issues in all domains between pre and post-intervention: Bringing up (18.3% pre; vs. 45.2% post; *p* = .001^**^) Coordinating care (19.7% pre; vs. 57.1% post; *p* < .001^**^) Provision of sexual health during the diagnosis/treatment (19% pre vs. 60% post; *p* < .0001^**^) and in the surveillance phase (23.2% pre vs. 51.6% post; *p* = .003^*^)
McBain L, [54]	Medical students	Confidence, comfort levels, and skills in performing a genital examination	Self–reported questionnaire Pre-post intervention and 1-year cohort	Skills, Confidence, and Comfort levels were improved (*p* < .001^**^), with higher range scores for female exam compared to a man
Ross MW, [47]	Nursing and midwifery students	Knowledge Communication skills Attitude	Self-reported survey	Knowledge and communication skills improved in all domains Attitudes toward anal sex and abortion were not changed after the intervention
Frasca K, [34]	Internal medicine residents	Behavioral change in sexual history taking	A qualitative method, self-reported assessment	6/8 participants applied the new skills to their clinical practice Five reported they planned to include sexual history-taking skills in future practice
White BP, [37]		Confidence Comfort preparedness	Self–reported assessment	Overall preparedness to provide comprehensive sexual health care improved from (*M* = 2.84, *SD* = 1.17) to (*M* = 4.37, *SD* = 0.49), CI 1.50 [1.03, 1.97], *p* < 0.5^*^) Comfort in initiating discussions about sexual health with patients improved from (*M* = 3.43, *SD* = 1.19) to (*M* = 4.40, *SD* = 0.89), CI 0.97 [ 0.45 – 1.48], *p* < 0.5^*^ Confidence providing sexual health improved from (*M* = 3.13, *SD* = 1.01) to (*M* = 4.40, *SD* = 0.68), CI 1.27 [0.88 – 1.66], *p* < 0.5^*^
Roth LT, [42]	Medicine residents	impact on clinical practice	Self–reported assessment, based on Kirkpatric Model	Clinical practice changes are reported in the following domains: 40% Asked about sexual identity (72% to 88.4%, *p* = .02) 40% asked about gender identity (23.3% to 43.5%, *p* = .02)
Mert – Karadas M, [51]	Nursing students	Communication skills and SSH taking	Online OSCE Students conducted individual interviews and recorded the sexual history taken	The study found an improvement in Effective communication skills median scores in the post-test (97.00 [87.00–99.00], *p* < .001^**^) compared to the pre-test (80.50 [64.00–92.00]) Reproductive SHH-taking skills improved from (70.52 [42.32–95.50]) in the pre-test to (93.51 [64.00–100.00], *p* < .001^**^) in the post-test

*Acronym: SHH* Sexual Health History, *OSCE* Objective Structured Clinical Examination, *M* Mean, *SD* Standard Deviation, *CI* Confidence Interval

^*^p < 0.05; ^**^p < 0.001

## Discussion

The current systematic review evaluated educational interventional studies that included SHE interventions for health professional students. Specifically, the review detailed the duration of these educational interventions with SHA content (knowledge, attitudes, and skills). Additionally, the review explored how the educational intervention was evaluated, whether through self-reported measures or via an actual performance of SHA. Among the 36 articles, which constituted an international health professional student sample, few educational interventions in SH were found for nursing students. If at all, these SHE interventions focused mainly on senior nursing students. This finding aligns with the literature review [19], wherein most educational interventions aimed at addressing SH include graduate students, interns, or professionals’ continuing education post-graduation.

The research findings corroborated the assertions made by Blakey and colleagues (2017) regarding the absence of comprehensive SH education for nursing students [10]. Therefore, the researchers argued that this lacuna in SHE contributes to the lack of emphasis on SH matters in professional practice [10]. Furthermore, these findings shed light on the global insufficiency of adequate SHE provided to health professional students during their early years of study, contradicting the recommendations set forth by the World Health Organization [6]. It is suggested that further research be conducted to examine the correlation between the omission of SHA in health professionals’ curricula, particularly in the early stages of learning, and practitioners’ inattentiveness to SH during clinical practice. Understanding that SHA is one of the essential competencies would encourage health professional education experts to integrate this content as suggested in the literature [9, 12, 13].

This systematic review identified various SHE interventions that differed in duration, educational content, and teaching methods. Similar variability was also observed in other systematic reviews [19, 20]. Coverdale (2011) emphasized the importance of consistency in determining educational variables and the need for diverse research designs to enhance the quality of evidence [19]. Our systematic review revealed differences in intervention variables and evaluation procedures. In addition, we noticed inconsistencies in research designs, as some studies lacked follow-up measurements, indicating the need for more rigorous evaluations in future studies. Similarly, our literature review uncovered diversity in the duration and content of educational interventions, consistent with the findings reported by Verrastro (2020) [20]. These variations underscored the lack of standardization in SHE for health professional students.

The literature suggested integrating SHA with all curricular components, including knowledge, attitudes, and skills [5, 18]. This integrated approach offered a foundational framework for comprehending the various factors that influence SH in both health and disease. By employing the three principles of SHA, students can acquire knowledge and skills, develop a deeper understanding of their personal attitudes toward the subject, and cultivate a sense of professional responsibility regarding SH as an integral part of their practice [5, 18]. It has been documented that professionals can only proceed to more advanced SH skills after reconciling personal beliefs with professional obligations [5, 18].

The current systematic review highlighted the variation in the content of educational intervention programs. For example, while many studies have developed interventions focused on the SH of LGBTQ populations and/or women’s health issues, only a few interventions have incorporated SHA skills based on the comprehensive 6P’S model [15] and the PLISSIT model [16]. This is unfortunate since these models provide a structured approach to discussing SH. Omitting these principles in most interventions or providing educational interventions that only cover specific aspects of SH may not foster a sense of comprehensive responsibility and dedication to the subject. Despite having positive attitudes toward the topic, students struggled to address patients’ SH, as reported in the literature, while SHA was perceived as daunting [11]. It is possible that establishing teaching standards for comprehensive SHA could provide a consistent foundation for professionals’ sexual health education. Nevertheless, it is advisable to conduct future research to determine whether this standard fosters the health professional students’ ability to initiate conversations about SH.

Finally, this systematic review reported different evaluation methods used to assess the effectiveness of SHE interventions. Only a few interventions included evaluating SHA in clinical practice. Instead, most of the programs relied on self-reported data, which may be affected by participant biases. As a result, it is challenging to draw definitive conclusions about the actual effectiveness of these interventions in practice. To address this issue, researchers recommend using an Objective Structured Clinical Examination (OSCE) for practical application, which has been identified as the most reliable way to evaluate curriculum [18].

### Limitations

This systematic review had several limitations. First, valuable insights from similar studies published in other languages were missed by solely incorporating articles in English. This fact possibly limited the quality of evidence and the extent to which the findings can be generalized. Moreover, it is conceivable that some pertinent studies were omitted from our review due to using a predetermined search strategy. Furthermore, it is worth noting that only a limited number of studies centered on nursing students, suggesting potential biases in interpreting the results. The present systematic review did not explore cultural and socio-demographic factors that could impact students’ attitudes and clinical abilities in SHA. Adapting programs requires tailoring interventions to cultural nuances and integrating content on cultural sensitivity within the curriculum.

## Conclusions

Health assessment encompasses a thorough approach that enables the evaluation of patient’s health from a biopsychosocial standpoint [5]. This approach considers various aspects of an individual’s life that can influence their well-being, including SH. Creating standardized SH education curricula and integrating vital SHA skills enables health professional students to acquire the necessary knowledge and capabilities for patient SH evaluation. This study highlighted a significant gap in SH educational intervention. Varied learning objectives, intervention durations, and curriculum methods posed challenges in evaluating intervention program effectiveness. Examining educational outcomes and establishing guidelines for comprehensive SH professional education, incorporating SHA skills, and extending it to all healthcare students involved in direct patient care, is crucial.

## Data Availability

The datasets used and analyzed during the current study available from the corresponding author (NB^1^) on reasonable request.
